# Effect of mineral and organic fertilizer on N dynamics upon erosion-induced topsoil dilution

**DOI:** 10.1016/j.heliyon.2024.e34822

**Published:** 2024-07-19

**Authors:** Isabel Zentgraf, Mathias Hoffmann, Jürgen Augustin, Caroline Buchen-Tschiskale, Sara Hoferer, Maire Holz

**Affiliations:** a Leibniz Center for Agricultural Landscape Research (ZALF) e.V., Group of Isotope Biogeochemistry and Gas Fluxes, Eberswalder Str. 84, 15374, Müncheberg, Germany; b Humboldt-Universität zu Berlin, Thaer-Institute of Agricultural and Horticultural Sciences, Invalidenstraße 42, 10099, Berlin, Germany; c Thünen Institute of Climate-Smart Agriculture, Federal Research Institute for Rural Areas, Forestry and Fisheries, Bundesallee 65, 38116, Braunschweig, Germany

**Keywords:** ^15^N labelingN recovery, Topsoil dilution, Soil erosion, Canola, Soil type

## Abstract

Erosion-induced topsoil dilution strongly affects cropland biogeochemistry and is associated with a negative effect on soil health and crop productivity. While its impact on soil C cycling has been widely recognized, there is little information about its impact on soil N cycling and N fertilizer dynamics. Here, we studied three factors potentially influencing N cycling and N fertilizer dynamics in cropping systems, namely: 1.) soil type, 2.) erosion-induced topsoil dilution and 3.) N fertilizer form, in a full-factorial pot experiment using canola plants. We studied three erosion affected soil types (Luvisol, eroded Luvisol, calcaric Regosol) and performed topsoil dilution in all three soils by admixing 20 % of the respective subsoil into its topsoil. N fertilizer dynamics were investigated using either mineral (calcium ammonium nitrate) or organic (biogas digestate) fertilizer, labeled with ^15^N. The fertilizer ^15^N recovery and the distribution of the fertilizer N in different soil fractions was quantified after plant maturity. Fertilizer N dynamics and utilization were influenced by all three factors investigated. ^15^N recovery in the plant-soil system was higher and fertilizer N utilization was lower in the treatments with diluted topsoil than in the non-diluted controls. Similarly, plants of the organic fertilizer N treatments took up significantly less fertilizer N in comparison to mineral fertilizer treatments. Both topsoil dilution and organic fertilizer application promoted ^15^N recovery and N accumulation in the soil fractions, with strong differences between soil types. Our study reveals an innovative insight: topsoil dilution due to soil erosion has a negligible impact on N cycling and dynamics in the plant-soil system. The crucial factors influencing these processes are found to be the choice of fertilizer form and the specific soil type. Recognizing these aspects is essential for a precise and comprehensive assessment of the environmental continuum, emphasizing the novelty of our findings.

## Highlights

• N fertilizer form and soil type greatly influence soil N turnover and storage.
• Negligible effect of topsoil dilution on soil N retention in coarse textured soil.
• Topsoil dilution led to fertilizer N incorporation into c- and fPOM, contrary to our hypothesis.

## Graphical abstract

**Figure:**
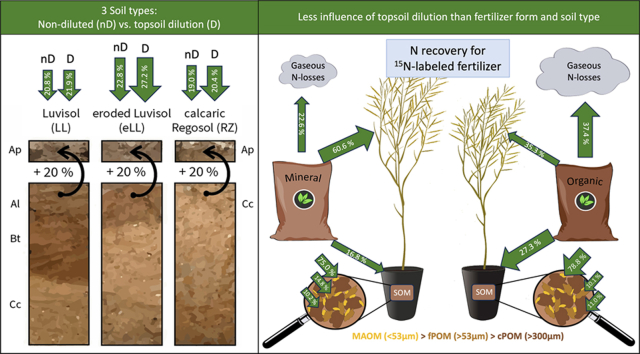

Ndff: Nitrogen derived from fertilizer
LL: Luvisol
eLL: Eroded Luvisol
RZ: Calcaric Regosol
DON: Dissolved organic nitrogen
TIC: Total inorganic carbon
TOC: Total organic carbon
cPOM: Coarse particulate organic matter
fPOM: Fine particulate organic matter
MAOM: Mineral associated organic matter
WHC: Water holding capacity
RCF: Relative centrifugal force

## 1 Introduction

Soil erosion is a major threat to soil fertility, causing an annual loss of 35.9 billion tons of topsoil [1]. This loss significantly impacts the elemental cycles especially in agricultural soils, resulting in a major reduction of crop yields, estimated at 0.4 percent per year [[2], [3], [4]].

Erosion-induced topsoil dilution is defined as topsoil removal and subsequent subsoil admixture, resulting from soil tillage. Topsoil dilution impacts and alters the nutrient storage of soils, leading to a shift in carbon (C) and nitrogen (N) dynamics and to an increase in the element's spatial variability [5,6]. While topsoil removal reduces crop productivity and soil health [7,8], there are also indications that erosion-induced topsoil dilution might increase N storage as a result of so-called N undersaturation [9].

Whereas a growing number of studies have investigated the effects of erosion on C cycling [[10], [11], [12], [13], [14]] little is known about the effects of erosion on N cycling and bioavailability [[15], [16], [17]]. Wang et al. [15] observed a clear enrichment in N in depositional soils throughout the soil profile, which was likely due to the increased macro-aggregate- and mineral-associated N. Those findings align with the research of Berhe and Torn [18] who found that eroded soils contain up to three times less N than depositional soils, with an increased spatial variability of N in eroded soils.

The impact of erosion and soil redistribution on N dynamics significantly affects both the turnover and efficient use of fertilizer N. Soil type and texture emerge as important factors with the potential to strongly influence these processes [19]. Recently, Li et al. [20], highlighted significant differences in N transformation based on soil type even under equivalent fertilization conditions. Their research underscored the critical role of soil organic carbon (SOC) and clay content in N storage and transformation. Hence, the analysis of both clay content and different fractions of SOM (particulate organic matter (POM) and mineral-associated organic matter (MAOM)) is essential for a better understanding of N dynamics in erosion-induced diluted topsoil [21]. These findings emphasize the need to adjust N management strategies according to soil types in order to control N losses and to achieve the best possible crop yields. However, predicting the impact of soil type on N dynamics in eroded soils, which show great heterogeneity and a rapid transition between various soil types within a small area [6], remains challenging [19,22,23].

Intensive agriculture strongly depends on high amounts of N fertilizers, however the production and use of synthetic N fertilizers through the Haber-Bosch process has a strong environmental impact and high energy demand [24,25]. Alternatively, organic fertilizers like animal slurries and biogas digestate can be used to help reduce energy consumption and improve soil fertility and crop performance [26,27]. Digestate not only contributes to resource recovery [28] but also contains a high amount of nutrients and is acknowledged to have a positive effect on N availability and crop yields [29]. Additionally, organic fertilizer may lead to higher N sequestration, and the incorporation of digestate after conventional tillage may have the potential to reduce its negative environmental impact and restore soil fertility [30,31]. However, the inaccurate use of these by-products can also lead to ecological problems such as increased N leaching into water bodies and increased greenhouse gas emissions [[32], [33], [34]]. Moreover, different fertilizer forms have various and often opposed effects on soil N cycling [35,36]. At present, the combined effect of soil type, erosion and different fertilizer forms on soil N transformation and plant N usage is little understood and needs further investigation. This knowledge gap underscores the need for research, particularly concerning the potential impact of increased fertilizer N fixation induced by topsoil dilution on plant N supply.

To fill this knowledge gap, we conducted a pot experiment under controlled conditions investigating the influence of soil type, topsoil dilution and fertilizer form on soil and fertilizer N dynamics. Canola was grown until full maturity and fertilized with either organic (biogas digestate) or mineral (calcium ammonium nitrate, CAN) N. We used the ^15^N balance method to determine the fate of N from both fertilizers at the end of the experiment. This method allows for precise tracking and quantification of the fate of labeled N within the soil and for distinguishing between the fertilizer and SOM derived N within the plant.

Our primary objectives were twofold: first, to investigate how topsoil dilution across different soil types influences soil N retention and the utilization of N fertilizers; second, to examine the influence of N fertilizer forms on soil N recovery and its utilization. Additionally, we aimed to evaluate how these three factors, topsoil dilution, soil type and fertilizer form, impact the retention of N in soil organic matter fractions.

We hypothesized that.

a) Topsoil dilution leads to increased retention of fertilizer N in the soil, particularly in the MAOM fraction.
b) Soil types characterized by subsoils with elevated clay content possess a greater capacity for N fixation upon topsoil dilution compared to soil types with lower clay contents in the subsoil.
c) Mineral fertilized treatments show higher plant N use efficiency (NUE), while organic fertilized treatments show reduced N recovery in the plant but a greater retention of fertilizer N in the soil, particularly within the MAOM fraction.

## 2 Material and methods

### 2.1 Setup and preparation of pot experiment

We conducted a pot experiment with 12 treatments (3 x 2 x 2), each with four replicates consisting of three soil types with increasing erosion status, LL, eLL and RZ, two dilution states (diluted, which is indicated by a “D” in the following, and non-diluted topsoil, indicated by “nD”) and two N fertilizer forms (organic and mineral). Soils originated from the CarboZALF area in the AgroScapeLab Quillow landscape laboratory in the Uckermark region. Topsoil-dilution was achieved by mixing topsoil material with subsoil material of the respective soil type, at a ratio of 4:1, namely LL + Al (sandy), eLL + Bt (silty) and RZ + *Cc* (calcic). Soils were sieved to 2 mm prior to filling of pots. Subsoil material C and N content differed significantly by a factor of two to three. Soil characteristics of each soil type are shown in Table 1 and for each dilution treatment in Table. A1. Dry matter production, the mineral N forms within the soil, total N pools of plant and soil and state and fate of labeled ^15^N fertilizers were subsequently quantified. Fertilizer ^15^N recovery was calculated for plant and soil parts, including root, shoot, bulk soil, and soil organic matter (SOM), coarse particulate organic matter (cPOM), fine particulate organic matter (fPOM), and mineral-associated organic matter (MAOM).

**Table 1:** Soil characteristics of the three topsoils and three subsoils used in the experiment. Soil texture, total nitrogen (TN), total inorganic carbon (TIC), and total organic carbon (TOC) are given. Data provided by Prof. M. Sommer.

Soil type	Horizon	Depth (cm)	Sand (%)	Silt (%)	Clay (%)	pH (CaCl_2_)	TN (%)	C:N	TIC (%)	TOC (%)
Luvisol	Ap	0–7	64,7	27,7	7,7	6,5	0,07	10	0,3	0,7
eroded Luvisol	Ap	0–8	60,7	26,3	13,0	6,8	0,09	10	0,3	0,9
Calcaric Regosol	Ap	0–8	62,0	28,0	10,0	7,2	0,08	10	3,4	0,8
Subsoil	Al	30–50	62,0	32,0	6,0	5,1	0,02	7	<0.1	0,2
Subsoil	Bt	100–120	54,0	27,0	18,0	6,4	0,03	6	<0.1	0,2
Subsoil	*Cc*	160–200	55,0	31,0	14,0	7,4	0,02	4	11,6	0,1

The N fertilizer application rate for both N fertilizer treatments was set to 170 kg N ha^−1^ (567.5 mg N pot^−1^). For ^15^N labeling, ^15^N-labeled ammonium-nitrate (^15^NO_3_–^15^NH_4,_ atom% 5) was added to the mineral fertilizer (Thomaskali PK 8–20 (+3 MgO), Nordland Agrar GmbH, Süderlügum) in the mineral N fertilizer treatment. The ^15^NO_3_–^15^NH_4_ fertilizer was mixed directly with 80 ml of distilled water, where mineral fertilizer was dissolved, before filling the pots. To balance nutrients in the mineral fertilized treatments, 0.13 g kg^−1^ of calcium carbonate (CaCO_3_) was added to the soil to mimic CAN fertilizer addition close to field application. For labeling of the organic N fertilizer treatment, 2.55 g^15^N-labeled urea (atom% 62.75) was mixed with 98.9 g pot^−1^ biogas digestate 48 h before pot filling to ensure homogeneous labeling of the NH_4_^+^-N pool of the digestate. The biogas digestate consisted of cattle slurry and energy crop conferment and originated from Pflanzenbauhof GbR (Uckerland). Labeling (atom% 10.78) was restricted to the easily available mineral NH_4_^+^ –N pool [37], which is recognized as the primary source contributing to N uptake and N losses following slurry application [38,39]. Consequently, our ^15^N data primarily reflect the transformation of the digestate NH_4_^+^-N pool. The characteristics of the digestate and CAN used are shown in Table 2. The digestate was analyzed by AGROLAB Agrarzentrum GmbH, Germany, in March 2021.

**Table 2:** Characteristics of the organic (digestate) and mineral fertilizer (calcium ammonium nitrate, CAN) used in the experiment.

Fertilizer	WC (%)	TN (%)	NH_4_^+^-N (%)	P_2_O_5_ (%)	K_2_O (%)	MgO (%)	P (%)	S (%)	C/N	OM (%)
Digestate	92	7.2	3.5	1.7	9.5	0.9	0.4	0.5	6.3	78.1
CAN	/	27	/	8.0	20.0	3.0	3.5	0.0	/	0.0

In total, 48 plastic pots, consisting of grey pipes (HAT DN 125) with a volume of 2,7 cm^3^ were prepared. Pots were bottom closed to prevent leaching losses. Each pot received a total of 3.74 kg of soil to achieve a bulk density of 1.4 g cm^−3^. Either organic or mineral fertilizer as well as the specific amount of deionized water to reach 60 % water holding capacity (WHC) to ensure optimal plant growth were mixed in the soil homogeneously before filling. Mixing was carried out with a handheld mixer for 2 min each to ensure homogeneous fertilizer distribution. After filling of pots, 3 canola seeds (CAMPINO, *Brassica napus*) were placed 2 cm deep in the soil. Pots were placed randomized in a climate chamber (Fitotron HGC 1514, Weiss Technik GmbH, Reiskirchen. Germany) with a 12-12-h (16-8-h) day-night rhythm and temperatures ranging from 9 to 16 °C (night) 9–21 °C (day) depending on the specific growth stage of the canola. Average light intensity was 400 μmol m^−2^ s^−1^ for a period of 12 (16) hours. During emergence, two excess shoots were removed according to size and appearance leaving one sprout in each pot. Canola plants were cultivated until full maturity of the rapeseed grains (average of 140 days). During the vegetation period, watering took place every 2 days by manually weighting each pot and addition of the lost water to ensure a WHC of 60 %.

### 2.2 Shoot sampling and analysis

Minerally fertilized pots were harvested after 137 days and organic fertilized pots after 144 days. Plants were measured for plant height and cut at ground level to determine fresh mass. Shoots were dried at 58 °C for 48 h, weighed for dry matter (DM) content and milled for further analysis. Total carbon (TC) and total nitrogen (TN) were determined by dry combustion using an elemental analyzer (CNS928-MLC, Leco Instruments GmbH, Mönchengladbach, Germany, DIN ISO 13878; 10694). Analyses of isotope ^15^N values in soil and plant samples were determined by a Costech Elemental Analyzer (Conflo III, Thermo Electron GmbH, Bremen, Germany) at the University of Göttingen. After harvest, all pots (including soil and roots) were frozen at −20 °C until further processing.

### 2.3 Root sampling and analysis

After thawing, the main root in each pot was separated from bulk soil and transferred to a small bucket for further processing. Small and fine roots were picked by hand with tweezers for a standardized time of 15 min. Rhizosphere soil, defined as soil attached to the main root and the hand-picked roots, was separated by gentle hand washing. For this, an aliquot of 500 ml deionized water was added to the root samples from each pot and gently soaked and brushed to remove soil from the roots. The resulting suspension was dried at 60 °C for 48 h and weighed to determine the amount of rhizosphere soil. Root biomass was analyzed by drying the wet root samples at 40 °C for 24 h. Dried root samples were milled and analyzed for TC, TN and ^15^N enrichment.

### 2.4 Soil sampling and analysis

An aliquot of 50 g fresh soil was taken from each pot to analyze labeled and unlabeled mineral N at the beginning and the end of the experiment. Soil for mineral N (N_min_ = sum of NH_4_^+^-N plus NO_3_^−^-N) at harvest was taken before freezing the pots. N_min_ determination was done by the central laboratory at ZALF according to ISO 14256 with a CFA-SAN (Skalar analytic GmbH, Breda, Netherlands). Labeled ^15^NH_4_^+^-N and ^15^NO_3_^−^-N was analyzed via SPIN-MIRMS [40] at the University of Göttingen. TC, total inorganic carbon (TIC) and total organic carbon (TOC) were analyzed by the central laboratory at ZALF according to ISO 10694 with a multiphase determinator (RC 612, Leco Instruments GmbH, Mönchengladbach, Germany).

**Fig. 1:**
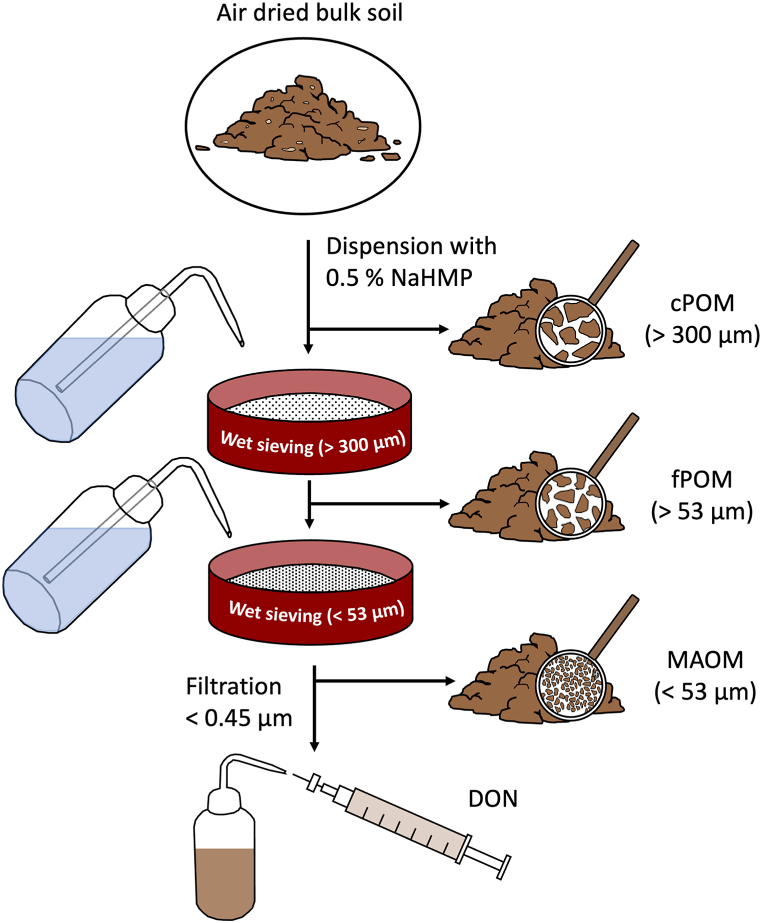
Particle size fractionation scheme. cPOM = coarse particulate organic matter; fPOM = fine particulate organic matter; MAOM = mineral-associated organic matter; DON = dissolved organic nitrogen.

Soil fractionation was done based on particle sizes (Fig. 1). The fractionation method was simplified based on other physical fractionation methods [41,42] and distinguished coarse particulate organic matter (cPOM, >300 μm), fine POM (fPOM, >53 μm) and mineral-associated organic matter (MAOM, <53 μm). 60 g bulk soil was mixed with 180 ml of 0.5 % sodium hexa metaphosphate (NaHMP) and 4.8 g glass beads (2.85–3.45 mm, Carl ROTH) and shaken on a mechanical shaker at 150 rpm for 18 h for aggregate dispersal. The suspension was sieved 3 times for 5 min over a >300 μm > 53 μm and <53 μm vibrating sieve tower. After every 5 min, the sieves were rinsed with 100 ml distilled water. After sieving, 40 ml from the <53 μm suspension were collected and centrifuged for 2 min. The supernatant was taken and analyzed for dissolved organic nitrogen (DON), and the remaining soil was returned to the <53 μm fraction to determine the total amount of MAOM. Each fraction was collected and dried at 60 °C for 72 h. After drying, soil was collected, milled at 85 rpm for 1 min with a ball mill and prepared for further analysis. Each of the three fractions was analyzed for TC and TN content as described in section 2.1. About 20 mg soil of each fraction was analyzed for ^15^N enrichment (see 2.2)

Microbial biomass was determined in bulk soil and rhizosphere soil through the chloroform fumigation method (CFE modified according to Brookes and Joergensen & Mueller [[43], [44]]. Per pot, two times 5 g fresh soil were weighed into tubes for fumigation and non-fumigation analysis. For fumigation samples, soil was rewetted with 250 μl of deionized water, homogenized and placed in a desiccator. After fumigation, samples were removed and transferred into 50 ml tubes. Both fumigated and non-fumigated samples were mixed with 20 ml of 0.05 M K_2_SO_4_, shaken for 30 min and centrifuged at a relative centrifugal force (RCF) of 4700 G. The supernatant was stored frozen at −20 °C until further analysis. TN within the samples was determined using a Total Nitrogen Analyzer (Mitsubishi TN-100, A1 Envirotech, Düsseldorf, Germany) equipped with an Auto Liquid Sampler and Auto Sample Injector (ASI-100, Dionex Softron GmbH, Germering, Germany). TC and TIC were analyzed in a 1:4 v/v extract dilution with double distilled water (Dimatoc 2000; DIMATEC Analysentechnik GmbH). The microbial N pool (mg kg^−1^ dry soil) was calculated as (ENf – ENnf)/kEN, where ENf = total N extracted from fumigated sample, ENnf = total N extracted from non-fumigated sample, where kEN = 0.54 [44].

### 2.5 Calculation of fertilizer N in plant and soil

For all calculations, background ^15^N was set to the natural abundance level of 0.3663 atom%. The atom% ^15^N excess of each sample indicates the ^15^N abundance of the sample subtracted by the natural abundance. Calculations of N parts derived from fertilizer (Ndff) in biomass and soil were calculated according to the following equation [45]:

Eq. (1) Ndff(%)=atom%Nexcessbiomass15atom%N15excessfertilizer*100

Assuming that fertilizer and soil N are the only sources of N for the plant, N derived from soil (Ndfs) can be expressed as the following:

Eq. (2) Ndfs(%)=100−Ndff(%)

For absolute values, Ndff (mg pot^−1^) was calculated according to Caste et al. [46] and Trivelin et al. [47] as follows:

Eq. (3) $$Ndff\;\left(mg\;{pot}^{-1}\right)=\frac{Ndff\left(\%\right)}{100}*total\;N$$

where total N corresponds to the total N content (mg pot^−1^) of each plant or soil part.

Fertilizer N recovery, defined as fertilizer N found at the end of the experiment was calculated according to Ref. [48]:

Eq. (4) NRec15(%)=Ndff(mgpot−1)NR(mgpot−1)*100

where NR (mg pot ^−1^) refers to the N rate fertilized in each pot [46,47].

Fertilizer N utilization (FNU) (%) was calculated as follows [45]:

Eq. (5) $$FNU\;\left(\%\right)=\frac{Fertilizer\;N\;yield\;\left(mg\;{pot}^{-1}\right)}{NR\;\left(mg\;{pot}^{-1}\right)}*100$$

where fertilizer N yield (mg pot^−1^) is calculated as the total N yield (mg pot^−1^) multiplied by the division of Ndff (%) by 100. N yield (mg pot^−1^) refers to the multiplication of the plant DM (mg pot^−1^) by its respective N concentration divided by 100.

N_min_ pool change was calculated as the difference of N_min_ value at the beginning of the experiment (t_0_) minus N_min_ values at crop harvest (t_harvest_).

### 2.6 Data treatment and statistical analysis

Calculations and analyses were performed using Excel 2022 and R (V. 2022.12.0.353; R Core Team 2022). Differences between treatments were tested by a three-way analysis of variance (ANOVA) using soil type, topsoil dilution and N fertilizer form as factors. For comparison within each factor, a one-way ANOVA was computed. Significant differences indicated by the ANOVA were checked by an LSD (Least significant distance) test for three-way comparison. Level of significance was set to p < 0.05. Visual analysis was performed to ensure normal distribution and variance homogeneity of residuals. All values are presented as means ± standard deviation (SD). To estimate the effect size of the three factors, eta squared (η^2^) was calculated when the ANOVA evaluated the effect as significant (p < 0.05) [49]. Values range from 0 to 1, with values approaching 1 signifying a greater proportion of variance that can be clarified by a specific variable within the model.

## 3 Results

### 3.1 Plant biomass, N uptake and fertilizer N utilization

Dry matter (DM) production until full maturity of canola differed between the two N fertilizer forms and topsoil dilution treatments (Table 3). Soil type showed significant differences in shoot DM for organic fertilized treatments between eLL-LL and LL-RZ, although eLL-RZ did not differ significantly. In general, organic fertilized treatments showed lower aboveground DM when compared to mineral fertilized treatments. Mineral N fertilized LL showed the highest shoot (47.2 g) and root (4.55 g) DM while the lowest shoot and root DM production was observed for organic fertilized, diluted eLL (eLL-D). Over all treatments shoot and root DM decreased with dilution and in the order LL > eLL > RZ, except for the LL soil type, in which root DM showed a contrary trend with higher DM in the diluted treatment. Shoot DM was affected most by N fertilizer form (η^2^ = 0.71), whereas root DM was predominantly affected by the soil type (η^2^ = 0.32).

**Table 3:** Root and shoot biomass production, N uptake by plant and fertilizer N utilization (FNU). Values shown are mean of treatment replicates ± SD (n = 4). Letters indicate significant differences between the treatments based on the three-way ANOVA.

Treatment		Root	Shoot	Plant N uptake	FNU
		DM (g pot^−1^)	DM (g pot^−1^)	TN (mg pot^−1^)	(%)
mineral	LL	4.6 ± 0.5^abc^	47.2 ± 2.2^a^	499.3 ± 35.6^bc^	50.6 ± 8.9^b^
	LL-D	5.4 ± 0.5^a^	46.3 ± 2.2^ab^	524.5 ± 8.7^abc^	55.0 ± 2.3^b^
	eLL	4.7 ± 0.5^abc^	42.4 ± 2.0^bc^	453.3 ± 13.6^cd^	50.5 ± 2.9^b^
	eLL-D	4.0 ± 0.3^abc^	38.2 ± 2.4^de^	390.0 ± 39.7^de^	47.1 ± 9.6^bc^
	RZ	4.5 ± 0.4^abc^	43.0 ± 2.5^bc^	624.7 ± 27.8^a^	76.0 ± 4.9^a^
	RZ-D	4.3 ± 0.4^abc^	41.9 ± 1.6^cd^	574.6 ± 25.0^ab^	59.0 ± 6.5^b^
organic	LL	5.0 ± 1.5^ab^	37.2 ± 2.3^e^	335.7 ± 27.3^ef^	32.6 ± 6.8^cd^
	LL-D	5.5 ± 1.4^a^	34.2 ± 1.8^ef^	311.2 ± 24.4^efg^	34.8 ± 5.0^cd^
	eLL	4.3 ± 0.7^abc^	30.3 ± 0.8^fg^	213.2 ± 20.8^gh^	25.0 ± 4.8^d^
	eLL-D	3.1 ± 0.6^c^	25.3 ± 0.7^h^	185.2 ± 23.1^h^	23.7 ± 6.4^d^
	RZ	3.4 ± 0.4^bc^	27.9 ± 0.9^gh^	243.1 ± 13.4^fgh^	32.0 ± 3.3^cd^
	RZ-D	3.4 ± 0.5^bc^	25.5 ± 1.4h	204.8 ± 31.4^gh^	30.0 ± 10.3^d^

Plant N uptake was significantly higher in the mineral fertilized treatments compared to the organic fertilized treatment (Table 3). In organic fertilized treatments N uptake decreased in the order LL > eLL > RZ and with topsoil dilution. For mineral fertilizer application, the treatment RZ displayed higher N uptake in comparison to LL and eLL. No significant differences in N uptake were observed between non-diluted and diluted treatments. The effect size for plant N uptake was greatest for the N fertilizer form (η^2^ = 0.74).

For FNU a significant fertilizer effect was observed with higher N use in mineral compared to organic fertilized treatments. No significant differences were found for FNU between the dilution treatments, apart from RZ mineral. Fertilizer form had the highest effect on FNU (η^2^ = 0.67).

### 3.2 ^15^N recovery

**Fig. 2:**
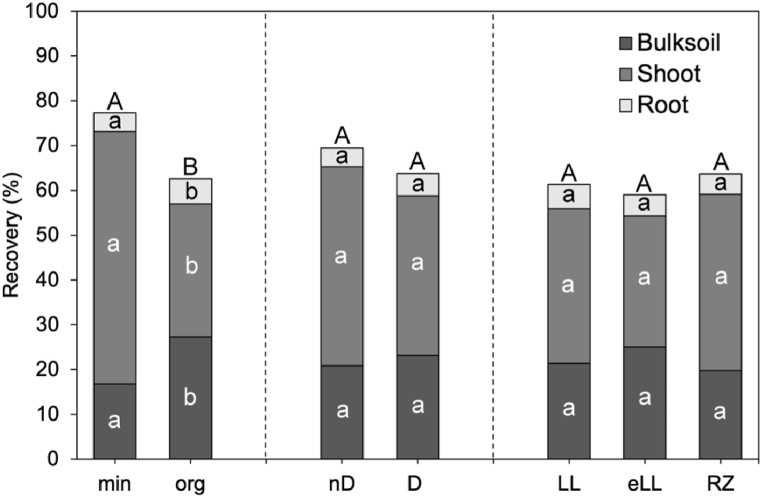
^15^N recovery of bulk soil, shoot and root in % of all treatments as average of each factor. Values shown are mean of treatment replicates. Abbreviations are min = mineral fertilization, org = organic fertilization, nD = non diluted, D = diluted; LL, eLL, RZ = indicating the specific soil type. Abbreviations stay the same through all graphs. Letters indicate significant differences between the treatments and within factors are based on one-way ANOVA.

Highest ^15^N recoveries were found for shoots (23.7–75.9 %), followed by bulk soil (14.3–35.6 %) and root (3.39 %–7.05 %) (Fig. 2). No significant differences in N recoveries among the soil types were found. N recoveries were greatest in non-diluted, mineral fertilized treatments (77.3 %), while organic fertilized treatments showed significantly lower recoveries (59.7–66.3 %). Organic fertilized treatments displayed an overall higher recovery in bulk soil and roots and lower recovery in shoots compared to mineral fertilized treatments. Undiscovered parts (100 % minus total ^15^N recovery) varied between 5.01 % (RZ mineral) and 40.24 % (eLL organic). In General, ^15^N recovery increased with dilution, although the differences were not significant. Effect sizes for ^15^N recovery in shoot and root followed the order fertilizer form > soil type > dilution (ns), which was equal for the ^15^N recovery in bulk soil (most affected by the fertilizer form, η^2^ = 0.7).

### 3.3 SOM-derived N and fertilizer-derived N in SOM fractions

**Fig. 3:**
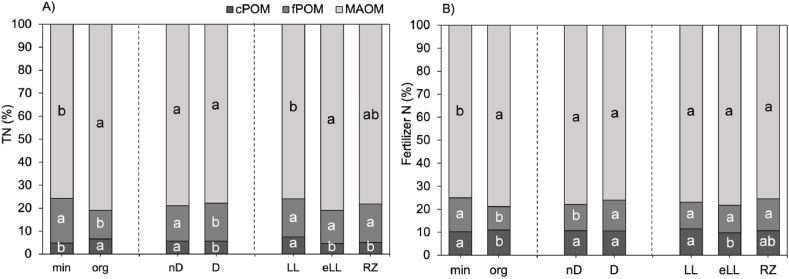
A) Relative distribution of bulk soil TN (%) into the three fractions for each factor and B) relative distribution of the fertilizer N (%) in the three fractions for each factor. Note that 100 % for Fertilizer N refers to share of fertilizer that was recovered in soil and not to the amount of fertilizer that was added at the beginning of the experiment. Values shown are mean of treatment replicates. Letters indicate significant differences between the treatments based on the one-way ANOVA.

Overall, the largest share of N was found in the MAOM fraction followed by the fPOM and the cPOM fraction, while the amount of N recovered within DON was much smaller compared to the N recovered in the solid fractions (Fig. 3A; Table. A3). Organic fertilization resulted in a relatively higher N storage within the cPOM and MAOM fraction compared to the mineral fertilization, while topsoil dilution led to an increased N storage within the cPOM and fPOM fraction (Fig. 3A). Soil type had little effect on N storage within the fractions. The LL showed the greatest N storage within the cPOM fraction, while the largest share of N was stored within the MAOM fraction for the eLL soil type (Fig. 3A). According to eta squared, fertilizer form had the strongest effect on N distribution within the fractions followed by soil type and topsoil dilution. Regarding the relative distribution of fertilizer N within the fractions, organic fertilization resulted in a higher relative fertilizer N recovery within the cPOM and MAOM fraction compared to the mineral fertilization (Fig. 3B). In contrast, topsoil dilution enhanced the relative fertilizer N recovery within the fPOM fraction but left the other fractions unchanged. Soil type had little effect on relative fertilizer N recovery, which was only increased in the cPOM fraction in the LL compared to the eLL soil type. According to eta squared, the relative distribution of the TN in the cPOM fraction was mostly affected by the soil type (η2 = 0.55), while the fPOM and MAOM fractions were most strongly affected by the fertilizer form (η2 = 0.68; 0.41 respectively).

**Fig. 4:**
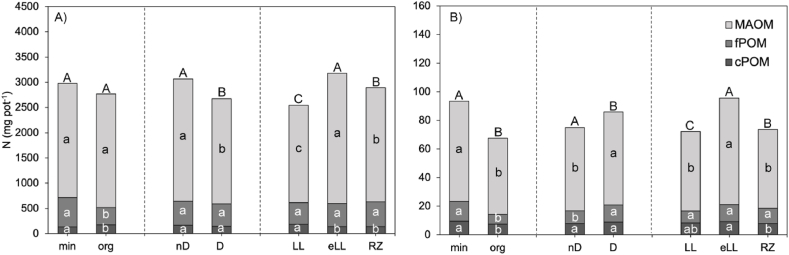
A) SOM-derived TN (mg pot^−1^) and B) fertilizer-derived N (mg pot^−1^) found in the different fractions of the soil at harvest as average of each factor. Values shown are mean of treatment replicates. Letters indicate significant differences between the treatments based on a one-way ANOVA.

The largest share of N recovered within the fractions was derived from SOM (2500–3000 mg N pot^−1^) (Fig. 4A), while much less fertilizer-derived N was recovered into the SOM fractions (70–100 mg N pot^−1^) (Fig. 4B).

Organic fertilization resulted in a reduced storage of SOM-derived N within the fPOM and cPOM fraction, while MAOM was not affected (Fig. 4A). Topsoil dilution had an opposite effect on the distribution of SOM-derived N within the fraction and resulted in a decreased storage of N within MAOM, while fPOM and cPOM remained unaffected (Fig. 4A). The distribution of SOM-derived N in the SOM fractions differed significantly with soil type in most cases. Lowest SOM-derived N was found in LL and highest in eLL (Fig. 3A). The cPOM fraction was greatest in LL and significantly smaller in eLL and RZ, while MAOM was distributed as follows: eLL > RZ > LL. The fPOM fraction did not show significant differences between the soil types. Based on the eta squared values, the distribution of SOM derived N in the cPOM and fPOM fractions was most strongly affected by the fertilizer form (η2 = 0.36; 0.75 respectively), while soil type had the highest effect on the MAOM fraction (η2 = 0.75).

Most fertilizer N was found in the MAOM fraction, followed by the fPOM and cPOM fractions (Fig. 4B). Most fertilizer N was recovered in the mineral treatment compared to the organic fertilized treatments. However, it needs to be considered that only the easily available N pool, such as urea or NH4+, was labeled in the digestate, so that the relative recovery of fertilizer N was higher for the organic fertilized treatment compared to the mineral treatment (mineral 32.9 % vs. organic 48.5 %). Overall, fertilizer-derived N recovery increased with topsoil dilution (Fig. 4B). Topsoil dilution did not affect the cPOM fraction but led to a significant increase in fPOM and MAOM.

Soil type did not affect the cPOM or the fPOM but had a strong effect on the fertilizer-derived N storage within MAOM, which followed the trend: eLL > RZ > LL. For the cPOM and fPOM fraction, the effect sizes according to eta squared were greatest for the fertilizer form (η2 = 0.26; 0.57 respectively). Conversely, the fertilizer-derived N in the MAOM fraction was most strongly affected by the soil type (η2 = 0.41).

## 4 Discussion

### 4.1 Impact of topsoil dilution on N recovery and the retention of N in different soil fractions

In this study, topsoil dilution was used to simulate the effect of erosion on three different soil types to assess its impact on fertilizer use and N cycling in canola. Based on the theory of N undersaturation in eroded soils [9] and subsequent dynamic replacement [50], we hypothesized that a) topsoil dilution will lead to increased retention of fertilizer N in the soil and b) that this effect is particularly strong for subsoils with high clay contents. These hypotheses were only partially confirmed by our study.

Overall, topsoil dilution resulted in a decrease of soil variables like total C and N, DON and C:N. These findings are in line with previous studies finding that topsoil removal results in a reduction of soil nutrient contents and storage [[51], [52], [53]]. Despite nutrients like P, K and Mg being initially adjusted for with mineral fertilization, above and belowground biomass production was reduced, which agrees with previous research reporting reduced yields on soils treated with topsoil removal, due to a decrease in available nutrients [[48], [54], [55], [56]].

Topsoil dilution resulted in a slight but not statistically significant increase in total fertilizer N retention in the soil (Fig. 2), which is contrary to our first hypothesis. This result may be linked to the slight differences in clay content, with a maximum variation of 1 %, between the non-diluted and diluted treatments (Table. A1). This minimal difference may have had a limited effect on N saturation and retention in the soil. Further research is needed to assess whether a stronger increase in clay content upon topsoil dilution will result in an increase of fertilizer N retention.

Examining the impact of topsoil dilution on N distribution within various SOM fractions allowed precise quantification of fertilizer-derived N accumulation in more stable (MAOM) vs. less stable (cPOM, fPOM) fractions. Srinivasan et al. [57] studied the effect of topsoil removal on particulate and mineral associated C fractions in a comparable experimental setup, finding significantly lower total organic N and non-particulate organic N (NPON, i.e., mineral-associated organic N) concentrations. Our findings concur, showing a 12.7 % decrease in total N concentrations in diluted treatments compared to non-diluted treatments, with MAOM-N and POM-N fractions reduced by 13.9 % and 8.7 %, respectively. This reduction aligns with overall total N decreases observed in previous studies [57,58].

Fertilizer N was preferentially incorporated in the fPOM and cPOM fractions, which is in strong contrast to the second part of our first hypothesis, based on which we would have expected a higher incorporation of fertilizer derived N in the MAOM fraction in the diluted treatments, compared to the non-diluted treatments. Given that POM primarily consists of lightweight, plant-derived fragments such as phenols and cellulose [[59], [60], [61]], the presence of fertilizer in the POM fraction might be caused by the decomposition of fine roots, which contain substantial amounts of ^15^N. This observation is supported by a notable 14.2 % increase in fertilizer N in the roots of the diluted treatments compared to the non-diluted controls (Fig. 2).

### 4.2 Impact of soil type on N recovery and the retention of N in different soil fractions

Our findings revealed that soil type had a much stronger effect on fertilizer N retention in soil than topsoil dilution. In contrast to the small variation in clay content between the topsoil and subsoil within a specific soil type, major differences in clay content were observed when comparing different soil types (Table 1). Clay content is well-established as a factor contributing to enhanced N sorption in soil [62], which might explain the more pronounced impact of soil type on fertilizer N retention.

Soil type eLL had the highest fertilizer recovery in bulk soil, followed by LL and RZ (Fig. 2). As the eLL was the soil with the highest clay content, this finding indicates that soil N retention is driven by clay contents (Table. A1), which possess a greater capacity for N fixation, e.g., in form of microbial necromass or NH_4_^+^ (Table. A4) [[63], [64], [65]]. Similarly, Ding et al. [19] observed lower fertilizer N recovery in sandy soils compared to loamy soils, supporting the results from our experiment. Contrary to this, the RZ soil type with lowest recovery in bulk soil had a medium sand content (62 %), compared to eLL (60.7 %) and LL (64.7 %). This result was probably superimposed by the high N uptake of the plants grown in this soil type and a subsequently high plant DM production. Under field conditions, the RZ studied is characterized by minimal soil development, a low WHC, shallowness and a susceptibility to erosion. This makes the RZ less favorable for agricultural use, prospecting low yields [66], which was also found by Vaidya et al. [67], who observed lowest yields on the RZ soil type compared to non- and slightly eroded soils. As the bulk density and water availability were controlled for in our experiment, canola may have had favorable conditions for growth in the RZ compared to field studies, resulting in high plant DM and N uptake in our study. This underlines the need for field studies to reflect realistic plant growth and soil conditions.

Total N retention and its distribution across different SOM fractions were influenced more by soil type than by dilution, due to the greater variability in N content between soil types. The MAOM fraction had the highest total N (78.34 %), followed by fPOM (15.96 %) and cPOM (5.70 %), aligning with other studies where up to 93 % of N was found in the MAOM fraction [15,18,19]. Fertilizer-derived N showed the same distribution pattern. These findings are consistent with studies [[68], [69], [70]] that demonstrated the MAOM fraction as a greater sink for 15N-labeled fertilizer compared to the POM fraction. Most fertilizer N was found in the eLL soil type, followed by RZ and LL, corresponding to their clay content and N fixation capacity, which decreases in the same sequence (Table 1).

We found that MAOM was the largest N sink for fertilizer N and, in absolute terms, fertilizer retention in MAOM increased in the diluted compared to the non-diluted treatment (Fig. 4B). However, when considering relative fertilizer N distribution within the fractions, we observed that fertilizer N was preferentially stored in the cPOM fraction after dilution (Fig. 3B). Fertilizer N recovery in the cPOM fraction was almost two times higher than the total N recovery in this fraction. Similar values were observed for all three soil types, where highest relative fertilizer concentration in the cPOM was found in the LL, followed by the RZ and the eLL, consistent with the change in sand content of the respective soil types. Once more, as POM is prominently plant-derived [[59], [60], [61]], it is likely that the fertilizer found in the cPOM fraction resulted from decomposed fine roots, containing high amounts of ^15^N, as suggested by Bosshard et al. [68] who also found higher fertilizer derived N in POM compared to MAOM fractions. Under organic fertilization, this may either be attributed to the incorporation of particulate organic N compounds into aggregates [71] or, under mineral fertilization, N may have entered the fraction through plant debris. Thus, it is possible that the fertilizer did not actually bind to the cPOM but rather that the increased recovery can be attributed to the fine roots and root debris that were recovered in that fraction.

In our coarse textured sandy-loam soils (Table. A1), POM may play a more significant role in storing fertilizer N than MAOM. This aligns with Huys et al. [72], who found that coarse-textured soils are crucial for C (and N) storage, as they efficiently store C in the POM pool, which unlike MAOM, lacks a saturation limit. Consequently, texture and soil type might be fundamental when considering (fertilizer)-N sequestration in soil. These findings moreover support the “whole-soil” (MAOM + POM) approach introduced by Barré et al. [73]. This concept emphasizes the inclusion of both MAOM and POM in the concept of C (N) storage in view of climate change mitigation, as POM represents a significant proportion of total SOC (SON) stocks [[74], [75], [76]].

### 4.3 Impact of fertilizer form on N fertilizer use efficiency

We hypothesized higher NUE in mineral-fertilized treatments and lower N recovery with greater soil retention in organic-fertilized treatments, indicating higher N losses. Those hypotheses were confirmed by our study. Since the more complex organic fraction of the digestate was unlabeled, the ^15^N data only reflect the transformation of the easily available N pool such as NH_4_^+^ or urea.

Previous research on long-term agricultural fields subjected to erosion has shown that soil N content is primarily influenced by the type and quantity of N fertilizer, with particularly pronounced effects in sandy soils [30,77]. This agrees with our study, where fertilizer form had the strongest impact on fertilizer N sequestration in soils (Table. A6) while the N fertilizer quantity was kept constant. Alongside changes in soil C and N, fertilizer form strongly affected shoot biomass production. Highest DM production was found in the mineral fertilized treatments compared to digestate fertilization (Table 3), although equal amount of N was applied. This is in contrast to some studies where, under equal fertilizer N conditions, plants fertilized with digestate showed comparable or even higher yield and DM production compared to mineral fertilized plants [[78], [79], [80], [81]]. However, it is notable that DM production strongly depends on the specific organic fertilizer and crop variety, which is why certain studies have also observed lower yields [82,83]. The comparable low DM production may be due to the large organic matter composition (78.1 %) (e.g., compared to 63 % [84]) of the digestate used in our study. In general, organic N may exhibit slower availability to plants compared to inorganic N, due to a slow mineralization rate [85]. This mineralization rate is significantly influenced by environmental factors such as soil moisture, pH and temperature, as well as the chemical composition of the organic material. Therefore, it is plausible that most of the N was initially bound within organic compounds and only became plant accessible post-mineralization. This process may account for the overall lower total N uptake observed in treatments with digestate application (Table 3).

Our findings revealed significantly higher fertilizer N losses in organic fertilized treatments (37.4 %) when compared to mineral fertilized treatments (22.6 %) (Fig. 2). Here, only the easily available organic N pool of the digestate was labeled, and the observed losses in the organic fertilized treatments therefore pertain specifically to this pool. These losses likely occurred in gaseous forms such as N_2_, N_2_O, NO or NH_3_, as our experimental setup effectively controlled for leaching losses. This imbalance may be attributed to the comparatively higher microbial denitrification rate (reduction of NO_3_^−^ to N_2_ [86]), which may be a consequence of the easily available mineral N in combination with high organic C and high microbial biomass C and N after organic fertilization (Table. A2, Table. A4). These factors favor denitrification rates and are in line with the findings of various authors [[87], [88], [89]], who described a clear correlation of increased soil C and N with increased microbial denitrification rates. Our results also align with a study conducted by Vaidya et al. [67] who observed the highest N_2_O emissions in organically fertilized treatments using the same soils employed in our experiment.

Moreover, the increased N losses in the organic fertilized treatment might occur in the form of ammonia (NH_3_) volatilization. This hypothesis is supported by relatively high pH values (>7) and fairly high moisture content (60 % WHC) during the whole growing period, favoring NH_3_ losses [90].

## 5 Conclusions

Our results indicate that N fertilizer form but also soil type play an overarching role for soil N turnover and NUE, while topsoil dilution showed a minor influence on these parameters. We therefore conclude that, at least for coarse textured soil types, topsoil dilution is of limited use for increasing C or N stocks. While our findings suggest a trend towards a decline of fertilizer use efficiency with topsoil dilution and an increase in fertilizer N retention in soil, additional research is required to establish this theory further.

In contrast to topsoil dilution, soil type strongly affected N cycling in our study, likely caused by the strong differences in texture, particularly clay content, and soil pH between the soil types. Hence, our conclusion emphasizes the critical importance of considering the great heterogeneity and swift transitions between different soil types in order to better understand N cycling and fertilizer N use in erosion affected agricultural landscapes.

The choice of N fertilizer form significantly influences both the utilization and transformation of fertilizer N. In our study, organic fertilizers promoted greater N sequestration but also resulted in higher N losses, resulting in a reduced FNU by crops. Consequently, these findings emphasize the need for careful consideration of N fertilizer choice and management practices to optimize FNU, while mitigating their negative environmental effects also on eroded croplands. In summary, our study elucidates the intricate relationships among nitrogen fertilizer form, soil type, and topsoil dilution, providing insights crucial for sustainable agricultural practices.

## Funding statement

This study was supported by the German 10.13039/501100005908Federal Ministry of Food and Agriculture (10.13039/501100010812FNR Grant: 22404117) within the research project “Krumensenke.”

## Data availability

The data that support the findings of this study are openly available through the BONARES repository at https://doi.org/10.4228/zalf-4vvc-d715.

## CRediT authorship contribution statement

**Isabel Zentgraf:** Writing – review & editing, Writing – original draft, Visualization, Project administration, Investigation, Formal analysis, Data curation. **Mathias Hoffmann:** Writing – review & editing, Writing – original draft, Visualization. **Jürgen Augustin:** Writing – review & editing, Writing – original draft, Resources, Methodology, Funding acquisition, Conceptualization. **Caroline Buchen-Tschiskale:** Writing – review & editing, Validation. **Sara Hoferer:** Investigation. **Maire Holz:** Writing – review & editing, Writing – original draft, Supervision, Resources, Formal analysis, Conceptualization.

## Declaration of competing interest

The authors declare the following financial interests/personal relationships which may be considered as potential competing interests: Isabel Zentgraf reports financial support was provided by 10.13039/501100010812Fachagentur Nachwachsende Rohstoffe eV. If there are other authors, they declare that they have no known competing financial interests or personal relationships that could have appeared to influence the work reported in this paper.

## Figures and Tables

**Table A1:** Soil texture and pH values of the control (non-diluted) and diluted treatments for each soil type and fertilization form. Data provided by Prof. M. Sommer.

Treatment	Dilution	% Sand	% Silt	% Clay	pH (CaCo3) mineral	pH (CaCo3) organic
LL	Non-diluted	64.7	27.7	7.7	7.1	7.1
LL + Al	Diluted	64.1	28.5	7.3	6.8	7.0
eLL	Non-diluted	60.7	26.3	13.0	7.3	7.3
eLL + Bt	Diluted	59.3	26.5	14.0	7.4	7.3
RZ	Non-diluted	62.0	28.0	10.0	7.5	7.5
RZ + *Cc*	Diluted	60.6	28.6	10.8	7.6	7.6

**Table A2:** Total soil nitrogen (TC), soil carbon (TC), soil organic Carbon (TOC) and C:N at harvest of the different treatments. Values shown are mean of treatment replicates ± standard deviation (n = 4). Letters indicate significant differences between the treatments based on the three-way ANOVA.

Treatment		TN %	TC	TOC %	C:N (−)
mineral	LL	0.08 ± 0.002^cd^	0.75 ± 0.015^e^	0.73 ± 0.032^de^	9 ± 0.29^cd^
	LL D	0.07 ± 0.001^e^	0.62 ± 0.011^f^	0.58 ± 0.009^h^	8 ± 0.18^e^
	eLL	0.08 ± 0.001^bc^	0.88 ± 0.006^d^	0.81 ± 0.022^bc^	10 ± 0.25^bc^
	eLL D	0.07 ± 0.001^ef^	0.76 ± 0.032^e^	0.69 ± 0.022^efg^	10 ± 0.42^b^
	RZ	0.07 ± 0.001^ef^	1.24 ± 0.009^b^	0.79 ± 0.031^bcd^	12 ± 0.43^a^
	RZ D	0.06 ± 0.002^g^	1.26 ± 0.028^b^	0.65 ± 0.027^fg^	11 ± 0.37^a^
organic	LL	0.08 ± 0.002^d^	0.75 ± 0.016^e^	0.75 ± 0.053^cde^	10 ± 0.49^bc^
	LL D	0.07 ± 0.001^f^	0.64 ± 0.022^f^	0.63 ± 0.025^gh^	10 ± 0.52^bcd^
	eLL	0.09 ± 0.002^a^	0.92 ± 0.032^c^	0.89 ± 0.021^a^	10 ± 0.10^bcd^
	eLL D	0.08 ± 0.002^b^	0.78 ± 0.011^de^	0.76 ± 0.012^cde^	9 ± 0.16^cd^
	RZ	0.09 ± 0.002^a^	1.26 ± 0.020^ab^	0.85 ± 0.029^ab^	10 ± 0.46^bcd^
	RZ D	0.08 ± 0.002^cd^	1.32 ± 0.028^a^	0.70 ± 0.25^ef^	9 ± 0.46^de^

**Table A3:** Soil N in the three soil fractions (cPOM, fPOM and MAOM) and dissolved organic nitrogen (DON) of the total soil. Values shown are mean of treatment replicates ± standard deviation (n = 4). Letters indicate significances between the treatments based on the three-way ANOVA. Note that DON values are given in mg kg^−1^.

Treatment		cPOM (>300 μm)	fPOM (>53 μm)	MAOM (<53 μm)	DON
		TN (g kg^−1^ cPOM)	TN (g kg^−1^ fPOM)	TN (g kg^−1^ MAOM)	TN (mg kg^−1)^
mineral	LL	0.30 ± 0.03^a^	0.26 ± 0.03^ef^	2.78 ± 0.6^a^	2.84 ± 0.9^abc^
	LL-D	0.23 ± 0.02^cd^	0.27 ± 0.01^de^	1.98 ± 0.2^abc^	2.54 ± 0.8^abc^
	eLL	0.22 ± 0.01^cd^	0.32 ± 0.04^cd^	2.71 ± 0.1^a^	2.42 ± 0.2^bc^
	eLL-D	0.19 ± 0.01^d^	0.36 ± 0.01^bc^	2.16 ± 0.1^abc^	2.29 ± 0.6^bc^
	RZ	0.23 ± 0.01^cd^	0.42 ± 0.03^a^	2.13 ± 0.2^abc^	2.50 ± 0.5^bc^
	RZ-D	0.20 ± 0.00^d^	0.39 ± 0.03^ab^	1.95 ± 0.3^abc^	1.85 ± 0.4^c^
organic	LL	0.32 ± 0.02^a^	0.21 ± 0.02^g^	2.62 ± 0.7^ab^	4.10 ± 0.6^a^
	LL-D	0.30 ± 0.02^a^	0.19 ± 0.01^g^	1.62 ± 0.1^c^	3.56 ± 0.34^ab^
	eLL	0.29 ± 0.02^ab^	0.22 ± 0.02^fg^	2.32 ± 0.1^abc^	3.84 ± 0.6^ab^
	eLL-D	0.30 ± 0.04^a^	0.22 ± 0.01^efg^	2.09 ± 0.2^abc^	3.10 ± 0.4^abc^
	RZ	0.29 ± 0.01^ab^	0.22 ± 0.02^fg^	2.16 ± 0.5^abc^	3.12 ± 0.5^abc^
	RZ-D	0.25 ± 0.01^bc^	0.20 ± 0.01^g^	1.75 ± 0.2^bc^	2.88 ± 0.9^abc^

**Table A4:** Microbial N (N_mic_), ammonium-N (NH_4_^+^ –N) and nitrate-N (NO_3_^−^ –N) at harvest. Values shown are mean of treatment replicates ± standard deviation (n = 4).

Treatment		N_mic_	NH_4_^+^-N	NO_3_^−^ –N
		mg kg^−1^	mg kg^−1^	mg kg^−1^
mineral	LL	22.2 ± 1.9^cd^	35.32 ± 4.0^cd^	45.42 ± 2.9^a^
	LL D	9.5 ± 4.7^d^	32.35 ± 1.0^def^	42.54 ± 0.8^ab^
	eLL	49.6 ± 7.5^ab^	32.70 ± 5.1^de^	37.89 ± 3.8^c^
	eLL D	26.4 ± 1.7^ab^	27.54 ± 1.2^ef^	38.75 ± 0.9^bc^
	RZ	46.3 ± 6.3^b^	25.72 ± 4.0^f^	32.00 ± 3.6^d^
	RZ D	60.2 ± 4.0^a^	28.10 ± 3.1^ef^	35.96 ± 1.7^cd^
organic	LL	55.9 ± 4.6^ab^	46.55 ± 1.8^a^	10.44 ± 0.3^d^
	LL D	54.1 ± 3.8^ab^	44.27 ± 1.3^ab^	9.27 ± 0.2^d^
	eLL	54.4 ± 6.2^ab^	44.24 ± 3.1^abc^	4.57 ± 0.1^f^
	eLL D	56.8 ± 4.3^ab^	33.93 ± 3.1^de^	4.15 ± 0.4^f^
	RZ	61.0 ± 8.9^a^	41.76 ± 2.1^bcd^	3.38 ± 0.2^f^
	RZ D	57.4 ± 5.4^ab^	38.16 ± 2.1^bcd^	3.46 ± 0.2^f^

**Table A5:** Mineral nitrogen (N_min_) values at time point t_0_ and t_harvest_ and calculated usage. Values shown are mean of treatment replicates ± standard deviation (n = 4).

Treatment		N_min_ (mg pot^−1^)		Usage
		t_0_	t_harvest_	Mg pot^−1^
mineral	LL	301.88 ± 26.1^a^	21.86 ± 2.8^a^	280.02^a^
	LL-D	280.05 ± 6.5^ab^	18.92 ± 2.5^abc^	261.12^ab^
	eLL	263.94 ± 33.4^abc^	16.46 ± 0.4^abc^	247.48^ab^
	eLL-D	247.85 ± 7.6^bcd^	17.82 ± 0.8^abc^	230.03^bc^
	RZ	215.81 ± 28.1^def^	19.53 ± 0.8^abc^	196.28^cde^
	RZ-D	239.54 ± 17.5^cde^	17.47 ± 0.3^abc^	222.07^bcd^
organic	LL	213.07 ± 7.3^def^	18.25 ± 3.3^abc^	194.82^cde^
	LL-D	200.16 ± 4.1^efg^	16.73 ± 3.7^abc^	183.43^def^
	eLL	182.48 ± 11.4^fgh^	19.98 ± 2.1^ab^	162.50^efg^
	eLL-D	142.39 ± 13.1^i^	15.40 ± 1.5^bc^	126.99^g^
	RZ	168.78 ± 8.7^ghi^	16.92 ± 1.7^abc^	151.86^fg^
	RZ-D	155.62 ± 7.4^hi^	14.23 ± 3.1^c^	141.39^g^

**Fig. A1:**
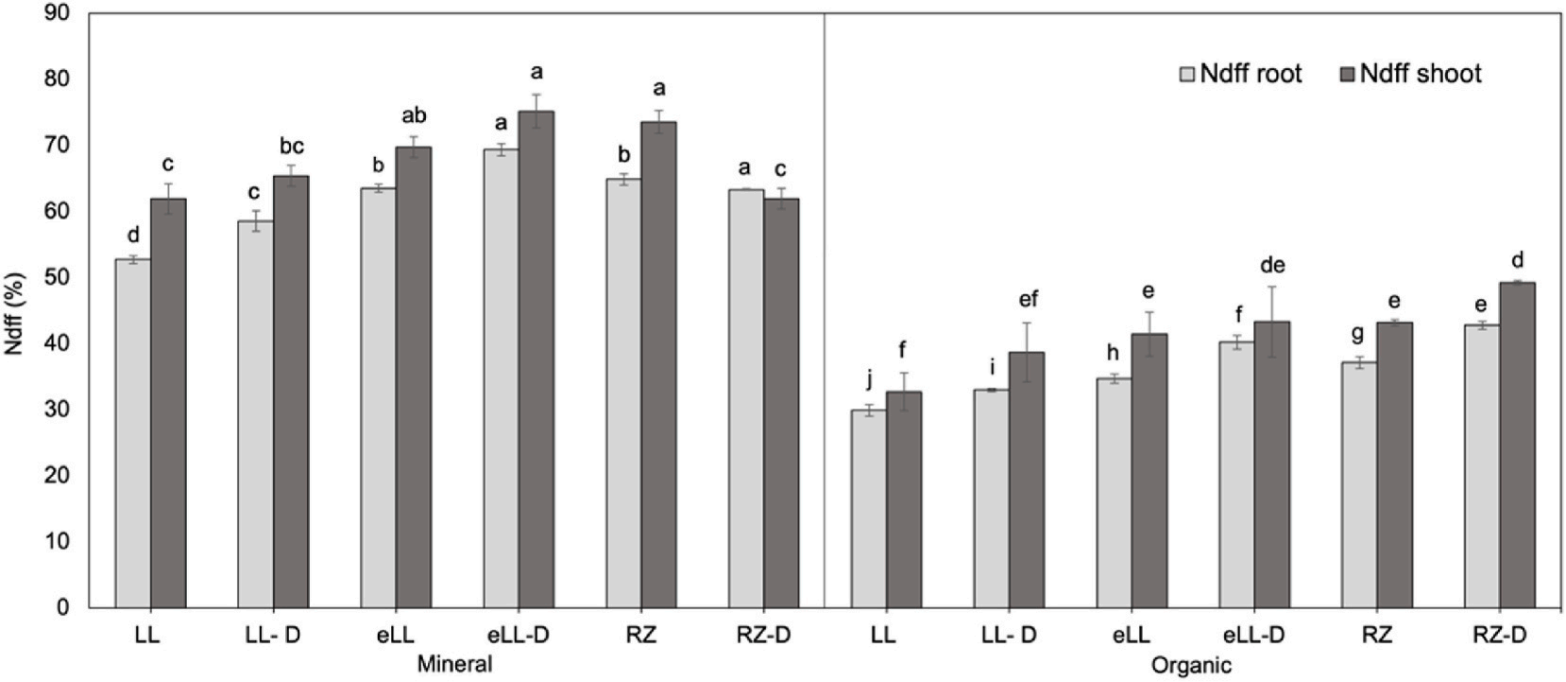
Nitrogen derived from fertilizer (Ndff) in plant biomass (root and shoot). Letters indicate significant differences between the treatments based on a three-way ANOVA. Error bars indicate the standard deviations of the mean (n = 4).

**Fig. A2:**
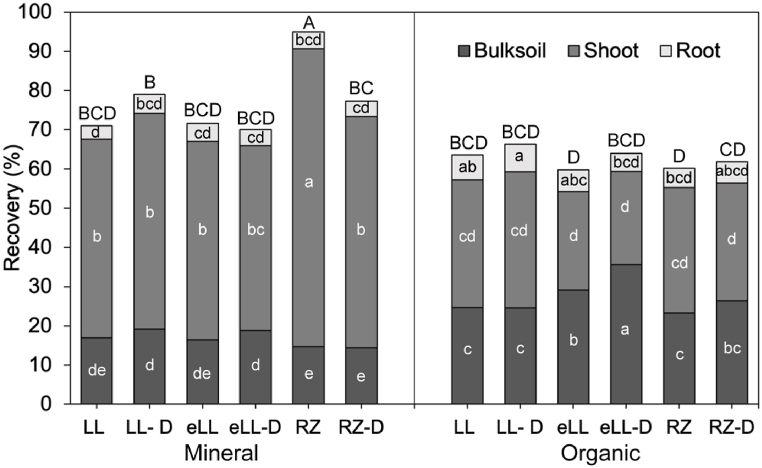
^15^N recovery of bulk soil, shoot and root in % of all treatments. Values shown are mean of treatment replicates (n = 4). Abbreviations are min = mineral fertilization, org = organic fertilization, nD = non diluted, D = diluted; LL, eLL, RZ = indicating the specific soil type. Letters indicate significant differences between the treatments and are based on a three-way ANOVA.

**Fig. A3:**
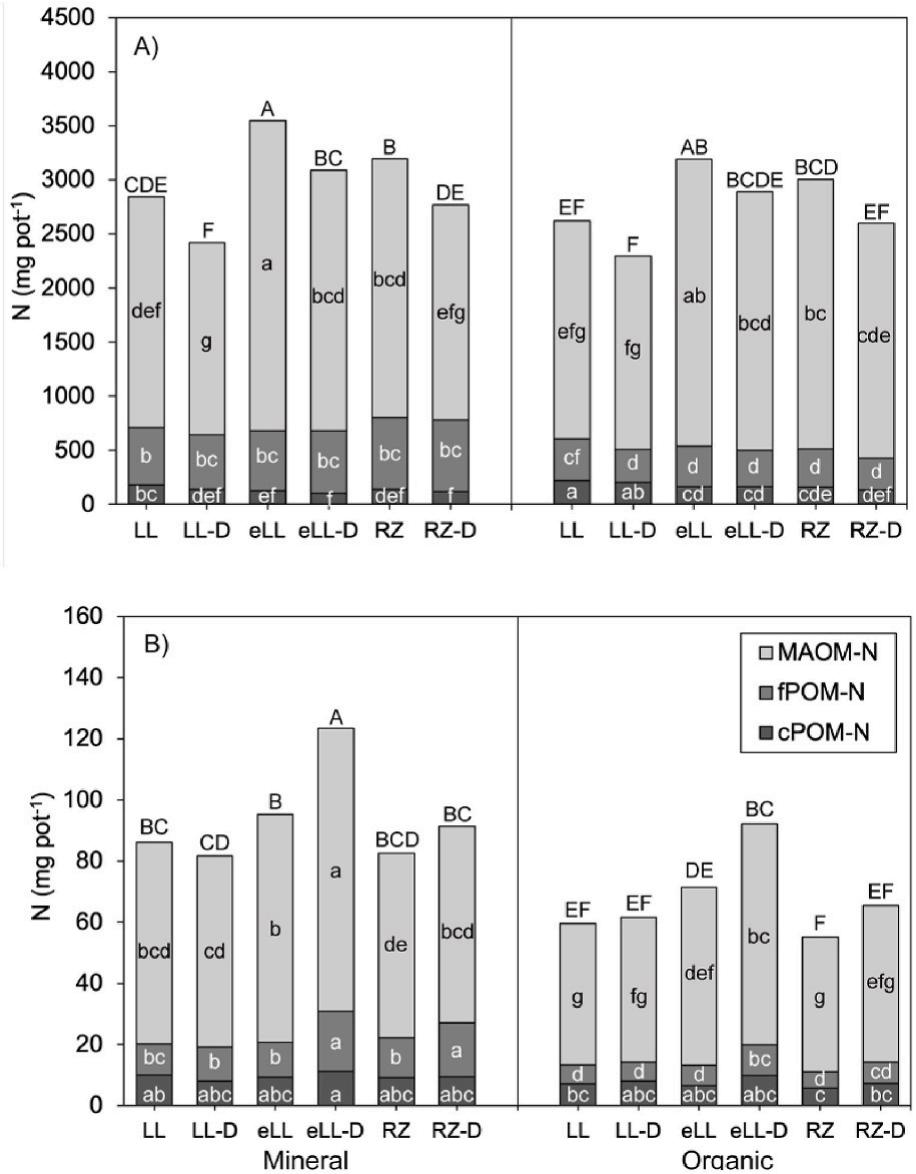
A) SOM derived TN (mg pot-1) and B) fertilizer derived N (mg pot-1) found in the different fractions of the soil at harvest. Values shown are mean of treatment replicates (n = 4). Letters indicate significant differences between the treatments based on the three-way ANOVA.

**Fig. A4:**
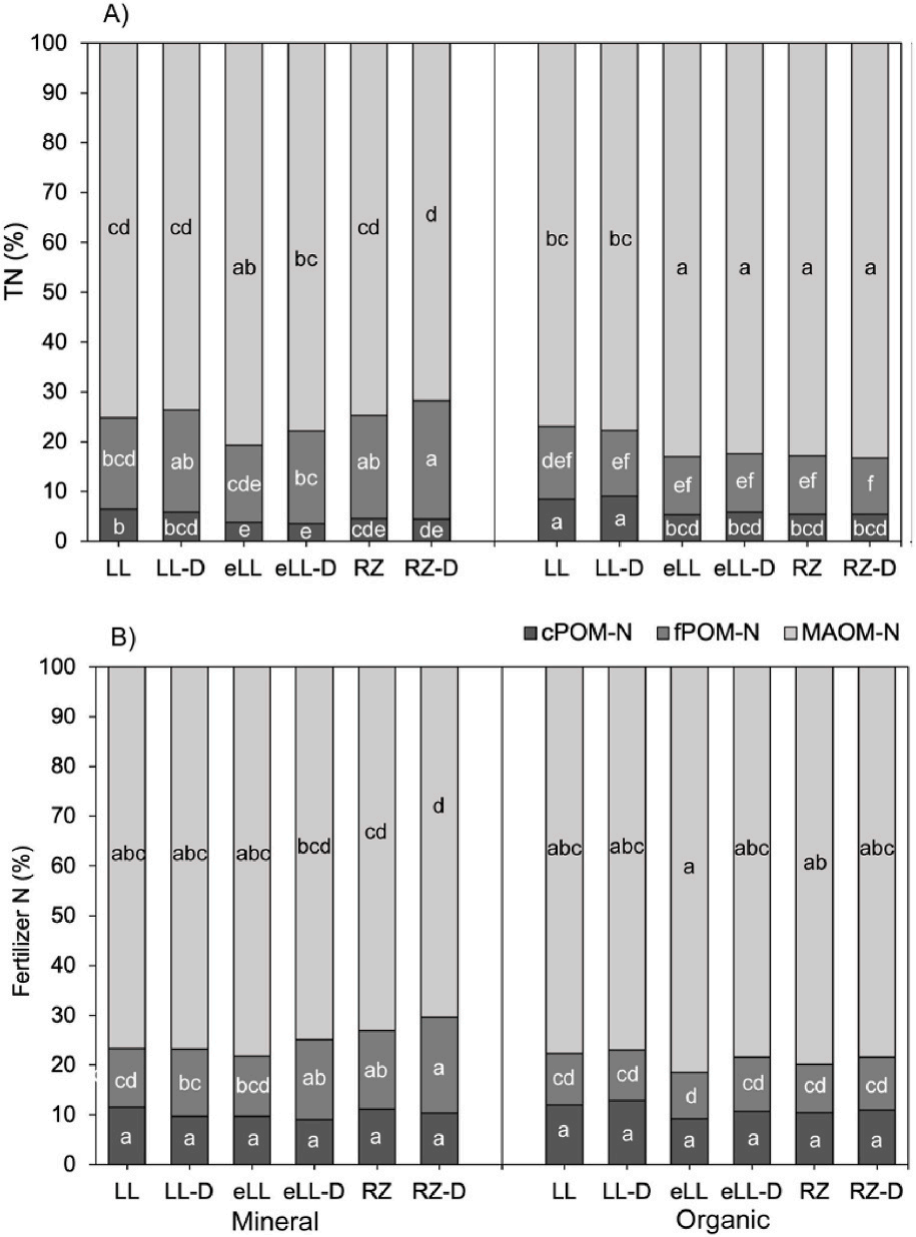
A) Relative distribution of bulk soil TN (%) into the three fractions for each factor and B) relative distribution of the fertilizer N (%) in bulk soil into the three fractions for each factor. Values shown are mean of treatment replicates (n = 4). Letters indicate significances between the treatments based on the three-way ANOVA.

**Table A6:** Eta squared (η^2^) values for tested variables.

Eta squared (η2)	Soil properties				Biomass and fertilizer N				Recovery			Fertilizer derived N			SOM derived N			Realtive Fertilizer and TN (%)					
Factor	TN	TC	TOC	C:N	RootDM	ShootDM	PlantNuptake	FU	RecoveryShoot	RecoveryRoot	RecoveryBS	15NcPOM	15NfPOM	15NMAOM	cPOM	fPOM	MAOM	15 N cPOM	15 N fPOM	15 N MAOM	TN cPOM	TN fPOM	TN MAOM
Soiltype	0,13	0,92	0,27	0,21	0,32	0,17	0,10	0,1	0,10	0,08	0,12	0,08	0,1	0,41	0,35	0,04	0,62	0,18	0,09	0,11	0,55	0,06	0,24
Fert.type	0,28	0,01	0,08	0,13	0,05	0,71	0,74	0,67	0,67	0,34	0,7	0,26	0,57	0,36	0,36	0,75	ns	ns	0,48	0,29	0,29	0,68	0,41
Dilution	0,30	0,02	0,55	0,03	0,004	0,03	0,01	ns	ns	ns	0,03	0,060	0,11	0,06	0,07	ns	0,22	ns	0,08	0,07	ns	0,015	0,18
Soiltype:Fert.type	0,25	ns	ns	0,44	ns	0,016	0,07	0,05	0,05	0,11	0,07	ns	0,04	ns	0,05	0,07	0,02	ns	0,1	0,16	0,04	0,08	0,13
Soiltype:Dilution	####	0,02	ns	ns	0,11	ns	ns	0,02	0,02	0,1	0,014	0,10	0,05	0,06	ns	ns	ns	ns	ns	ns	ns	ns	ns
Fert.type:Dilution	ns	ns	ns	ns	ns	ns	ns	ns	ns	ns	0,004	ns	0,02	ns	0,02	0,01	0,01	0,0800	0,03	ns	ns	0,03	0,02
Soiltype:Fert.type:Dilution	####	ns	ns	0,04	ns	ns	ns	ns	ns	ns	0,012	ns	ns	ns	ns	ns	ns	ns	ns	ns	ns	ns	ns
